# A ROR1 small molecule inhibitor (KAN0441571C) induced significant apoptosis of ibrutinib‐resistant ROR1^+^ CLL cells

**DOI:** 10.1002/jha2.232

**Published:** 2021-05-20

**Authors:** Amineh Ghaderi, Mohammad‐Ali Okhovat, Layung Sekar Sih Wikanthi, Ann Svensson, Marzia Palma, Johan Schultz, Thomas Olin, Anders Österborg, Håkan Mellstedt, Mohammad Hojjat‐Farsangi

**Affiliations:** ^1^ Department of Oncology‐Pathology BioClinicum, Karolinska Institutet Stockholm Sweden; ^2^ Department of Hematology Karolinska University Hospital Solna Stockholm Sweden; ^3^ Kancera AB, Karolinska Institute Science Park Solna Sweden

**Keywords:** BCL‐2 inhibitor, CLL, ibrutinib resistance, ROR1 inhibitor

## Abstract

ROR1 – a receptor tyrosine kinase – is overexpressed in CLL. Ibrutinib, a Bruton's tyrosine kinase inhibitor, is clinically effective in CLL but patients may develop resistance. We evaluated the effect of an ROR1 inhibitor, KAN0441571C, in CLL cells from six patients obtained before and after developing resistance to ibrutinib. The ROR1 inhibitor induced apoptosis in ibrutinib‐resistant CLL cells to the same degree as in ibrutinib‐sensitive cells and dephosphorylated ROR1. This was also noted in one patient who became resistant to both ibrutinib and the Bcl‐2 inhibitor venetoclax. The combination of ROR1 inhibitor and venetoclax had a synergistic apoptotic effect on ibrutinib‐resistant cells.

## 1 INTRODUCTION

The ROR1 receptor tyrosine kinase is expressed during embryogenesis but down‐regulated in the majority of normal adult tissues. However, ROR1 is expressed in various malignancies, which is of importance in the oncogenic process and of significance for cancer cell proliferation, survival and metastatic potential [1].

ROR1 expression in malignancies was initially noted in chronic lymphocytic leukemia (CLL) [2]. ROR1 could also be found in normal B‐cell precursors in the bone marrow at an intermediate stage of maturation, but early and late B‐cell precursors did not express ROR1 [1] suggesting that ROR1 targeted therapies may not affect normal B cells. High expression of ROR1 was related to enhanced constitutive activation of AKT and associated signaling molecules compared to cells with low ROR1 expression [3]. In CLL, high expression of activated ROR1 was associated with progressive disease [4]. An ROR1 small molecule inhibitor (SMI) could induce dephosphorylation of ROR1 in CLL cells as well as of canonical and non‐canonical Wnt signaling proteins with subsequent induction of apoptosis [5].

ROR1 has been shown to crosstalk with the B‐cell receptor (BCR) through the BCR complex and Bruton's tyrosine kinase (BTK) as well as with associated signaling molecules in acute lymphoblastic leukemia (ALL) B cells [3]. BTK is an important tyrosine kinase in the BCR signaling pathway driving the BCR signaling cascade leading to activation of downstream NF‐κB and phosphatidylinositol‐3‐kinase (PI3K) with a pro‐survival effect of CLL clone [6].

Ibrutinib is a covalent, irreversible SMI binding to BTK and highly effective for the treatment of CLL even in unselected (real‐world) patients [6]. The mechanism of action (MOA) is partly due to the ability to inhibit signal transduction through the BCR pathway. Even though most CLL patients initially respond to ibrutinib, an increasing number of patients develop resistance to the drug which is associated with mutation in the BTK gene at the ibrutinib binding site as C481S as well as mutations in the immediate downstream effector phospholipase Cγ2 (PLCγ2) (R665W and L845F) domain [7]. These mutations are detected in about 80% of CLL patients developing resistance to ibrutinib [8]. Thus, there is a great medical need to develop drugs with other MOA than ibrutinib. Venetoclax (a Bcl‐2 inhibitor) has been shown to be effective in ibrutinib‐resistant patients [9]. We have previously shown in preclinical models that small molecules inhibiting ROR1, KAN0439834 and KAN0441571C, induced tumor cell death in CLL and DLBCL cells which express activated ROR1 [5, 10].

In this study, tumor cell death in vitro after incubation with an ROR1 inhibitor (KAN0441571C) was compared in ROR1^+^ CLL cells obtained from patients before and after developing clinical resistance to ibrutinib. Venetoclax was used as a control as this drug may be effective in ibrutinib‐resistant cases. We also evaluated the combination of venetoclax and ROR1 inhibitor for induction of apoptosis in ibrutinib‐resistant cells. Moreover, a patient who had developed double‐refractoriness to both ibrutinib and venetoclax was also included.

## 2 PATIENTS AND METHODS

Patients were diagnosed and treated at the Department of Hematology, Karolinska University Hospital Solna, Stockholm, Sweden, according to the Swedish CLL guidelines. The study was approved by the National Ethics Authority (www.etikprovningsmyndigheten.se), and written informed consent was obtained from each patient before blood sampling. Six CLL patients‐ two females and four males, median age 67 years (range 50–85)‐ selected based on the availability of stored PBMC (liquid nitrogen) before and after developing clinical resistance to ibrutinib were included. Further two patients where only cell samples were available after developing clinical resistance to ibrutinib, were also enrolled. The Department of Clinical Genetics at the Karolinska hospital performed BTK mutation analyses by next generation sequencing (NGS). One of the patients also developed resistance to venetoclax in addition to ibrutinib (double‐refractory). The median proportion of BK mutated cells (second sample) was 36% (range 2%–50%). Apoptosis of PBMC (2 × 10^6^ cells/ml) in RPMI‐1640 was analyzed after incubation with the drugs for 24 h and stained for Annexin V/PI in flow‐cytometry [5]. ROR1 expression was determined by flow cytometry. Phosphorylation of ROR1 and BTK was evaluated by Western blot as previously described [5]. The effects in vitro on leukemic cell apoptosis (additive, synergistic or antagonist effects) after combination treatment with KAN0441571C and venetoclax was calculated by the Chou/Talalay method utilizing CompuSyn software (Combosyn Inc.) [11].

## 3 RESULTS AND DISCUSSION

**FIGURE 1:**
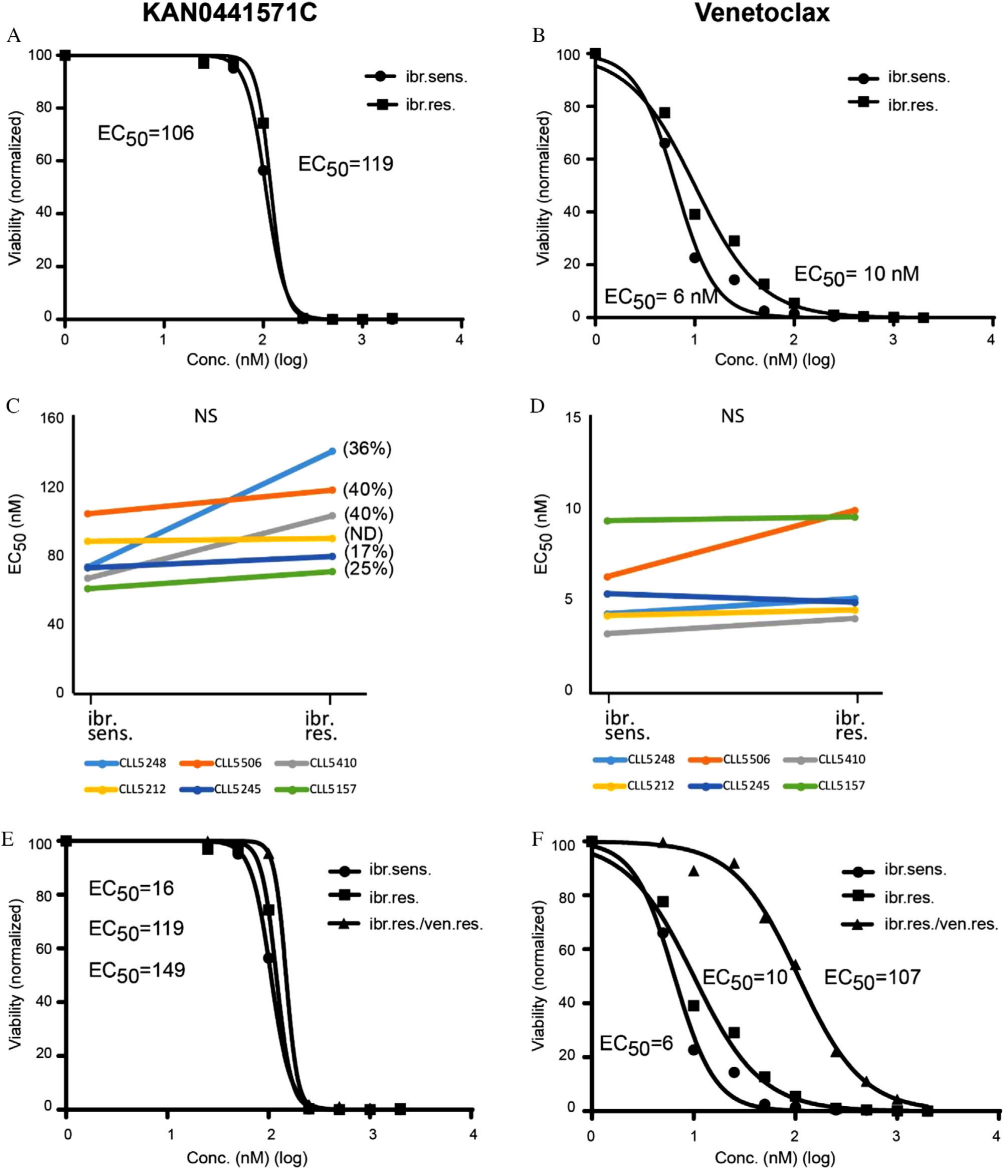
Apoptosis induced by a ROR1 SMI (KAN0441571C) in ibrutinib‐sensitive (sens) and ‐resistant (res) CLL. As a control and for comparison of apoptosis venetoclax is included. Annexin V/PI staining of leukemic cells was applied to determine the frequency of apoptotic cells after 24 h of incubation with the drugs. Dose‐response curves and EC_50_ values of (A) KAN0441571C and (B) venetoclax in a patient before and after developing clinical resistance to ibrutinib (40% BTK‐mutated cells). In (C) and (D), EC_50_ values for KAN0441571C and venetoclax in ibrutinib‐sensitive and ‐resistant CLL cells for each individual patients KAN0441571C are shown. Values within brackets indicate the frequency of BTK‐mutated cells. BTK‐mutated cells could not be detected (ND) in patient CLL 5232 when developing ibrutinib resistance. For statistical comparison a paired *t*‐test was used. (E) Dose‐response curves and EC_50_ values for KAN0441571C in ibrutinib‐sensitive and ‐resistant cells as well as in ibrutinib‐resistant/venetoclax‐resistant cells. (F) Dose‐response curves for venetoclax in the same cell populations as in E Abbreviation: NS, not significant.

KAN0441571C induced significant apoptosis to the same extent in both ibrutinib‐sensitive (BTK unmutated) and ibrutinib‐resistant (BTK‐mutated) ROR1^+^ CLL cells from the same patient. Representative dose‐response curves of a patient including EC_50_ values are shown in Figure 1A. The slope of the dose‐response curve was as expected for a drug with a pro‐apoptotic MOA. The apoptotic effect of venetoclax in the same patient is shown in Figure 1B. EC_50_ values for apoptosis of ibrutinib‐sensitive and ‐resistant CLL cells, respectively, incubated with KAN0441571C for all the individual patients are depicted in Figure 1C. There was no statistically significant difference between ibrutinib‐sensitive and ‐resistant cells in response to ROR1 inhibition. This was also noted for venetoclax treatment in vitro (Figure 1D) (Table S1). In ibrutinib‐resistant cases, there was a trend (*p* = 0.09) towards a high EC_50_ conc. of the ROR1 inhibitor for induction of apoptosis with increasing frequency of BTK‐mutated cells (Figure S1). No such correlation for the EC_50_ conc. of venetoclax was noted (data not shown).

One patient developed first clinical resistance to ibrutinib and then also to venetoclax. As can be seen in Figure 1E, the apoptotic response to the ROR1 inhibitor was similar in ibrutinib‐sensitive and ‐resistant cells as well as in double‐refractory cells. However, ibrutinib‐sensitive and ‐resistant cells responded equally well to venetoclax while double‐refractory cells required 10–15 times higher concentration of venetoclax to achieve the same apoptotic effect as compared to that required for ibrutinib‐sensitive and ‐resistant cells (Figure 1F).

Ibrutinib has been shown to have limited direct pro‐apoptotic activity in vitro and requires interfering with cross‐talk between CLL cells and the lymph node microenvironment. Advanced ex vivo drug testing systems are necessary [12]. Thus, the present ex vivo model does not allow evaluation of the apoptotic effect of ibrutinib.

**FIGURE 2:**
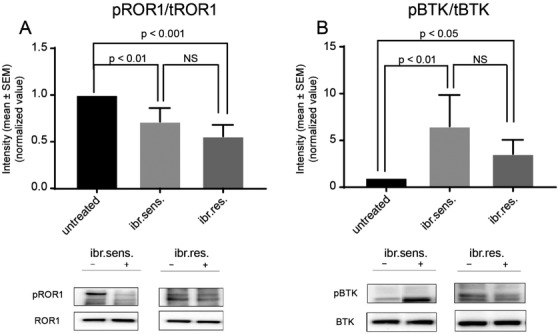
Phosphorylation of ROR1 and BTK in CLL cells (*n* = 4) sensitive and resistant to ibrutinib respectively after in vitro incubation (2 h) with an ROR1 inhibitor (KAN0441571C) at EC_50_ conc. (100 nM). Relative intensity of (A) pROR1/tROR1 and (B) pBTK/tBTK in relation to untreated cells (for statistical comparison a two‐sided paired *t*‐test was applied). At the bottom representative blots (pat. 5248) for each group are shown

KAN0441571C consistently dephosphorylated ROR1 in both ibrutinib‐sensitive and ‐resistant cells (Figure 2A) while the effect on BTK phosphorylation was varying but overall there was a statistically significant increase (Figure 2B). There was no significant difference between the effect on ibrutinib‐sensitive and ‐resistant cells for either ROR1 or BTK phosphorylation.

The combination of KAN0441571C and venetoclax showed a synergistic apoptotic effect in ibrutinib‐resistant CLL cells in all the six patients (Figure S2).

Crosstalk between the BCR complex and ROR1 signaling through activation of BTK, has been described in CLL, ALL, Burkitt's lymphoma and mantle cell lymphoma (MCL) [3, 13, 14]. In ALL cells, inhibition of pre‐BCR signaling by siRNA targeting BTK induced ROR1 upregulation, indicating communication between BTK and ROR1 signaling. Inhibition of one of these kinases was suggested to affect activation/phosphorylation of the other molecule, that is, if activation of one of the molecules was downregulated, activation of the other was upregulated as a compensatory mechanism for cell survival [3]. Our results in ibrutinib‐resistant cells are in line with those observations. The same results were seen in ibrutinib‐sensitive cells (data not shown). It has also been suggested that ROR1 modulates in a counterbalancing way pre‐BCR signaling including activation of AKT, ERK, MEK and survival of ALL cells [3].

KAN0441571C was equally effective in inducing apoptosis of CLL cells obtained from the same patients before and after development of ibrutinib‐resistance. The combination of KAN0441571C and venetoclax has previously been shown to significantly increase apoptosis of DLBCL cells in vitro compared to either drug alone [10]. These results were further corroborated in the present study showing that the combination of ROR1 inhibitor and venetoclax had a synergistic apoptotic effect on ibrutinib‐resistant cells. The mechanism underling the synergism between KAN0441571C and venetoclax is not clear. It is however interesting to note that in MCL targeting BTK by ibrutinib in combination with inhibition of either Bcl‐2 (venetoclax) or ROR1 (monoclonal antibodies) overcame drug resistance to ibrutinib. ROR1 inhibition by siRNA or monoclonal antibodies downregulated the NF‐kB p65 intracellular protein. Activation of the NF‐kB signaling pathway could antagonize ROR1 mediated apoptotic response [14]. The results are in line with our finding of a synergistic apoptotic effect in CLL cells by targeting both ROR1 and Bcl‐2 in ibrutinib‐resistant cells. We have also previously shown that the combination of ibrutinib and an ROR1 SMI (KAN0439834) augmented the killing of pancreatic cancer cells compared to either drug alone [15].

There is still a great medical need for novel drugs to be developed in CLL and also to be tested in combinations. Our results indicate that apoptosis can be induced by a ROR1 inhibitor in ibrutinib‐resistant CLL cells and preliminary also in venetoclax‐resistant cells. Furthermore, combination of ROR1 targeting drugs with ibrutinib or venetoclax seems to increase the tumor cell killing in vitro.

## Supporting information



Supporting Information

Supporting Information
