# Supplementary material for: A ROR1 small molecule inhibitor (KAN0441571C) induced significant apoptosis of ibrutinib‐resistant ROR1+ CLL cells
Source: EJHaem. 2021 May 20;2(3):498–502. doi: 10.1002/jha2.232 (PMC9176142; doi:10.1002/jha2.232)
Supplement: Supplementary file 1 — Supporting Information [file JHA2-2-498-s002.pptx]

## Slide 1
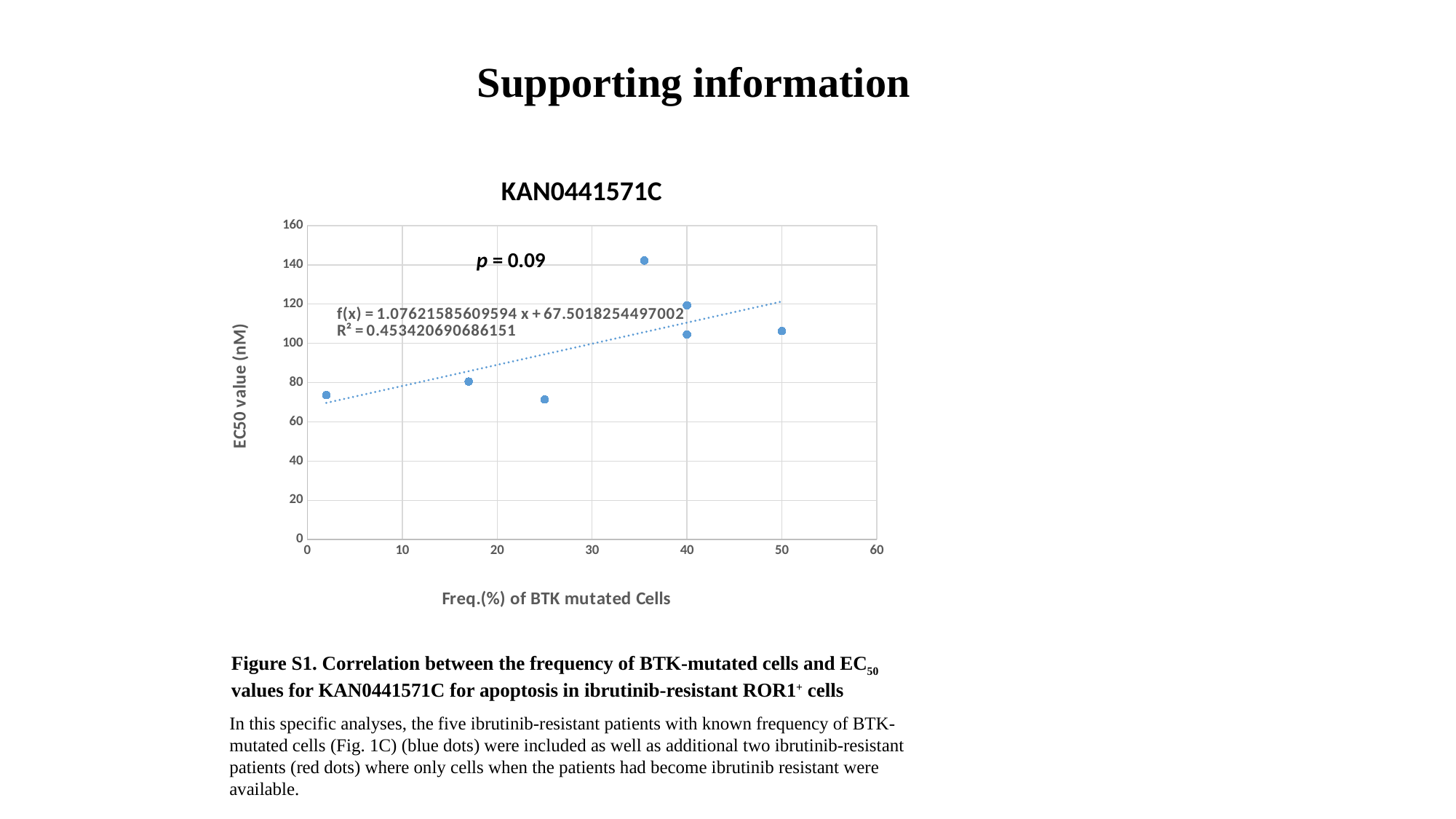

Supporting information
KAN0441571C
### Chart
| Category | EC50 KAN 571C |
|---|---|p = 0.09
Figure S1. Correlation between the frequency of BTK-mutated cells and EC50 values for KAN0441571C for apoptosis in ibrutinib-resistant ROR1+ cells
In this specific analyses, the five ibrutinib-resistant patients with known frequency of BTK-mutated cells (Fig. 1C) (blue dots) were included as well as additional two ibrutinib-resistant patients (red dots) where only cells when the patients had become ibrutinib resistant were available.

## Slide 2
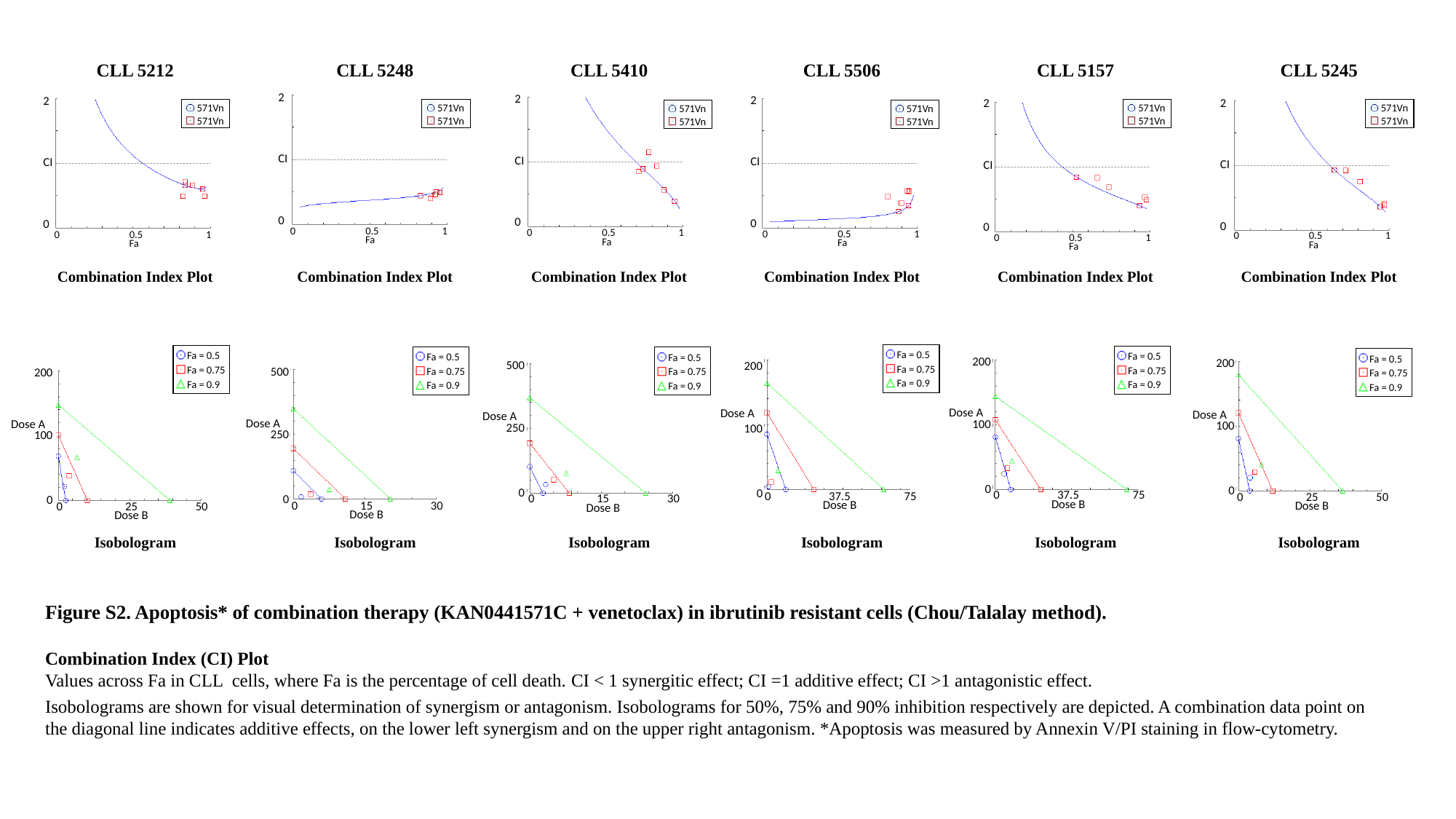

CLL 5157
CLL 5245
CLL 5212
CLL 5248
CLL 5410
CLL 5506
2
CI
0
2
CI
0
2
CI
0
2
CI
0
2
CI
0
2
CI
0
571Vn
571Vn
571Vn
571Vn
571Vn
571Vn
571Vn
571Vn
571Vn
571Vn
571Vn
571Vn
0	0.5	1
	Fa
0	0.5	1
	Fa
0	0.5	1
	Fa
0	0.5	1
	Fa
0	0.5	1
	Fa
0	0.5	1
	Fa
Combination Index Plot
Combination Index Plot
Combination Index Plot
Combination Index Plot
Combination Index Plot
Combination Index Plot
Fa = 0.5
Fa = 0.75
Fa = 0.9
Fa = 0.5
Fa = 0.75
Fa = 0.9
Fa = 0.5
Fa = 0.75
Fa = 0.9
Fa = 0.5
Fa = 0.75
Fa = 0.9
Fa = 0.5
Fa = 0.75
Fa = 0.9
Fa = 0.5
Fa = 0.75
Fa = 0.9
200
100
0
200
100
0
500
250
0
200
100
0
500
250
0
200
100
0
Dose A
Dose A
Dose A
Dose A
Dose A
Dose A
0	37.5	75
	Dose B
0	37.5	75
	Dose B
0	25	50
	Dose B
0	15	30
	Dose B
0	15	30
	Dose B
0	25	50
	Dose B
Isobologram
Isobologram
Isobologram
Isobologram
Isobologram
Isobologram
Figure S2. Apoptosis* of combination therapy (KAN0441571C + venetoclax) in ibrutinib resistant cells (Chou/Talalay method).
Combination Index (CI) Plot
Values across Fa in CLL cells, where Fa is the percentage of cell death. CI < 1 synergitic effect; CI =1 additive effect; CI >1 antagonistic effect.
Isobolograms are shown for visual determination of synergism or antagonism. Isobolograms for 50%, 75% and 90% inhibition respectively are depicted. A combination data point on the diagonal line indicates additive effects, on the lower left synergism and on the upper right antagonism. *Apoptosis was measured by Annexin V/PI staining in flow-cytometry.
