# Supplementary material for: A ROR1 small molecule inhibitor (KAN0441571C) induced significant apoptosis of ibrutinib‐resistant ROR1+ CLL cells
Source: EJHaem. 2021 May 20;2(3):498–502. doi: 10.1002/jha2.232 (PMC9176142; doi:10.1002/jha2.232)
Supplement: Supplementary file 2 — Supporting Information [file JHA2-2-498-s001.docx]

**TABLE S1** EC_50_ values for KAN0441571C and venetoclax in the same patient when sensitive and resistant respectively to treatment with ibrutinib.

|  | **KAN0441571C EC_50_ (nM)** | | | **Venetoclax EC_50_ (nM)** | | |
| --- | --- | --- | --- | --- | --- | --- |
|  | **Ibr-sens** | **Ibr-rest** | **Ratio**  **(Ibr-sens/Ibr-rest)** | **Ibr-sens** | **Ibr-rest** | **Ratio**  **(Ibr-sens/Ibr-rest)** |
| **Patient Code** |  |  |  |  |  |  |
| CLL5248 | 74.41 | 142.2 | 0.52 | 4.37 | 5.23 | 0.84 |
| CLL5506 | 105.6 | 119.4 | 0.88 | 6.4 | 10.04 | 0.64 |
| CLL5410 | 67.92 | 104.5 | 0.64 | 3.28 | 4.14 | 0.79 |
| CLL5212 | 89.36 | 90.93 | 0.98 | 4.3 | 4.6 | 0.93 |
| CLL5245 | 74.01 | 80.54 | 0.91 | 5.48 | 4.98 | 1.10 |
| CLL5157 | 61.85 | 71.39 | 0.86 | 9.47 | 9.68 | 0.98 |
